# Marine predator movements create seascape connectivity in remote coral reef ecosystems

**DOI:** 10.1186/s40462-025-00598-7

**Published:** 2025-10-10

**Authors:** Luciana C. Ferreira, Ronen Galaiduk, Ben Radford, Vinay Udyawer, Mark Meekan, Michele Thums, Rob Harcourt, Kate A. Lee, Eric A. Treml

**Affiliations:** 1 Australian Institute of Marine Science, Crawley, WA 6009 Australia; 2 Sharks Pacific, Rarotonga, Cook Islands; 3 UWA Oceans Institute, University of Western Australia, Crawley, WA Australia; 4 Ocean Sciences and Solutions Applied Research Institute, NEOM, Neom, Saudi Arabia; 5 School of Natural Sciences, Macquarie University, North Ryde, NSW 2113 Australia; 6 IMOS Animal Tagging, Sydney Institute of Marine Science, Mosman, NSW 2088 Australia

**Keywords:** Network analysis, Acoustic tracking, Habitat use, IMOS

## Abstract

**Background:**

Movement of marine predators can connect different habitats and create links that are key for maintaining metapopulation dynamics, genetic diversity, energy flow and trophic links within and between systems. This key ecological process is known as ecological connectivity.

**Methods:**

We used a combination of acoustic telemetry data, network analysis (graph theory), habitat modelling and machine learning methods to quantify movement patterns and habitat use of three coral reef predators (grey reef shark *Carcharhinus amblyrhynchos*, silvertip shark *Carcharhinus albimarginatus* and red bass *Lutjanus bohar*). We also assessed how movements and habitat preference influence connectivity in two remote reef systems (Rowley Shoals and Scott Reef) off Northwest Australia.

**Results:**

Grey reef shark movements created more substantial connections within reef systems, greater than silvertip sharks and red bass, with occasional long-ranging movement linking distant atolls. Core use areas (nodes with high degree centrality) were represented by low complexity habitats in shallow areas near passages in the reef crest, but varied among species, time of the day and sex. Overall, female sharks had larger networks with greater movement extent than males indicating potential sex-specific patterns in movement and connectivity of sharks at both local (within an atoll) and regional (within reef system) spatial scales. Red bass movements resulted in local-scale connectivity between the lagoon and nearby forereef areas, whereas reef shark connectivity operated at broader scales with movement along the forereef creating stronger connections across distant areas within the reef systems.

**Conclusions:**

The combination of animal tracking data, network analyses and machine learning allowed us to describe complex patterns of movement and habitat use within and between remote coral reef ecosystems and how they influence ecological connectivity over local and regional scales. Importantly, we suggest that the existing spatial protection across these remote coral reefs is effective in protecting the local-scale connectivity of mesopredators, yet broad-scale protection is required to effectively encompass the seascape connectivity of large predators which is crucial for the long-term health and stability of coral reef ecosystems.

**Supplementary Information:**

The online version contains supplementary material available at 10.1186/s40462-025-00598-7.

## Introduction

Movement is a major component in the life history of almost every organism [59]. There are many drivers (internal and external) of an animal’s decision to move [87], particularly in vertebrates that are capable of undertaking large-scale movements (100–1000s km). These movements connect different habitats, contributing to metapopulation dynamics, gene flow and trophic linkages [16, 71, 80, 110, 116]. The connection created by the movement of animals among habitats within ecosystems is a critical ecological process with system-wide implications [74]. However, the importance and effects of marine megafauna movement on system-level ecological connectivity has been largely overlooked.

Ecological connectivity is a consequence of not just the movement or dispersal of organisms between habitats, but a wide range of ecological processes, from structural relationships of the physical environment to the links formed by spatio-temporal biological and abiotic relationships within an ecosystem [16, 109]. Over the past 15 years the concept of ecological connectivity has gained increasing recognition as key information for the development and effectiveness of applied spatial management frameworks [1, 7, 13, 43, 65, 108]. For example, studies combining biophysical modelling, larval dispersal and network metrics have demonstrated the importance of functional connectivity for prioritisation in the design of potential marine reserve networks to maximise protection of critical habitats [8, 83].

Marine predators, such as sharks and large reef fishes, are a keystone element of the megafauna of coral reefs [23, 60, 94, 114]. They are capable of regulating the structure, composition and diversity of prey communities [5, 104] and their removal can affect the stability and resilience of ecosystems [49, 92, 94]. Overfishing is removing these predatory fauna from tropical habitats worldwide [60, 72, 114], while the reefs they inhabit are also afflicted by a multitude of anthropogenic disturbances including ocean warming [14], acidification [57], and high sediment flows from dredging activities and coastal development [33, 37]. For this reason, understanding the scale of ecological connectivity that marine predators drive is urgently required to manage and mitigate the impacts of their loss in coral reef systems.

Several remote atoll-like coral reefs exist on the edge of the continental shelf off Western Australia. These are relatively isolated from most anthropogenic impacts compared to nearshore environments [42] and long-term monitoring has shown that they have higher resilience to disturbances than coral reefs subject to chronic anthropogenic pressures [41, 42]. Earlier studies at these reef systems have assessed reef shark movements using acoustic telemetry [36], the effects of shark fishing and marine protected areas on shark and reef fish community structure and abundance [75, 76, 94, 102, 104], and trophic structure and interactions of mesopredators [4, 5]. Data collected from these remote systems offer an ideal opportunity for studies on the importance of movement and habitat preferences of large predators in creating and sustaining connectivity within and across coral reefs that are relatively unaffected by anthropogenic pressures. This can provide baseline information on habitat connectivity to inform management and conservation strategies for these species and ecosystems [2, 63, 79, 91].

A key analytical method for quantifying connectivity is network analysis, based on graph theory [78, 111]. A graph is a set of nodes (vertices) and links (edges) between nodes that indicates an ecological or functional connection, where additional attributes can be associated with nodes (spatial coordinates, habitat size and quality) and links (distance or weight) [111, 112] allowing numeric representation of links between habitats established by animal movements [61, 64, 80]. When applied to animal tracking data, networks describe movement steps between locations and can quantify system-wide movement dynamics while also accounting for the inherent autocorrelation of movement data [61, 64]. Node and edge metrics of movement networks can be used to identify central areas and key pathways, and to characterise emergent connectivity patterns within a system [111].

Here we used network analysis on acoustic tracking data of three coral reef predators (grey reef *Carcharhinus amblyrhynchos* and silvertip *Carcharhinus albimarginatus* sharks, and red bass *Lutjanus bohar*) combined with habitat modelling from two remote and isolated coral reef systems situated on the Northwest Shelf of Australia to improve our understanding of the movement patterns of reef predators and how they might affect connectivity at local (within individual atolls) and regional (within reef systems) spatial scales and how they vary between distant reef systems (~ 500 km apart). Specifically, we address i) how movement patterns vary among species and sites; ii) whether habitat attributes influence this movement; and iii) the spatio-temporal consistency in habitat use among species, sexes and reef systems. Finally, we highlight the ecological and management implications of the connectivity for coral reef predators.

## Methods

### Study sites

**Fig. 1:**
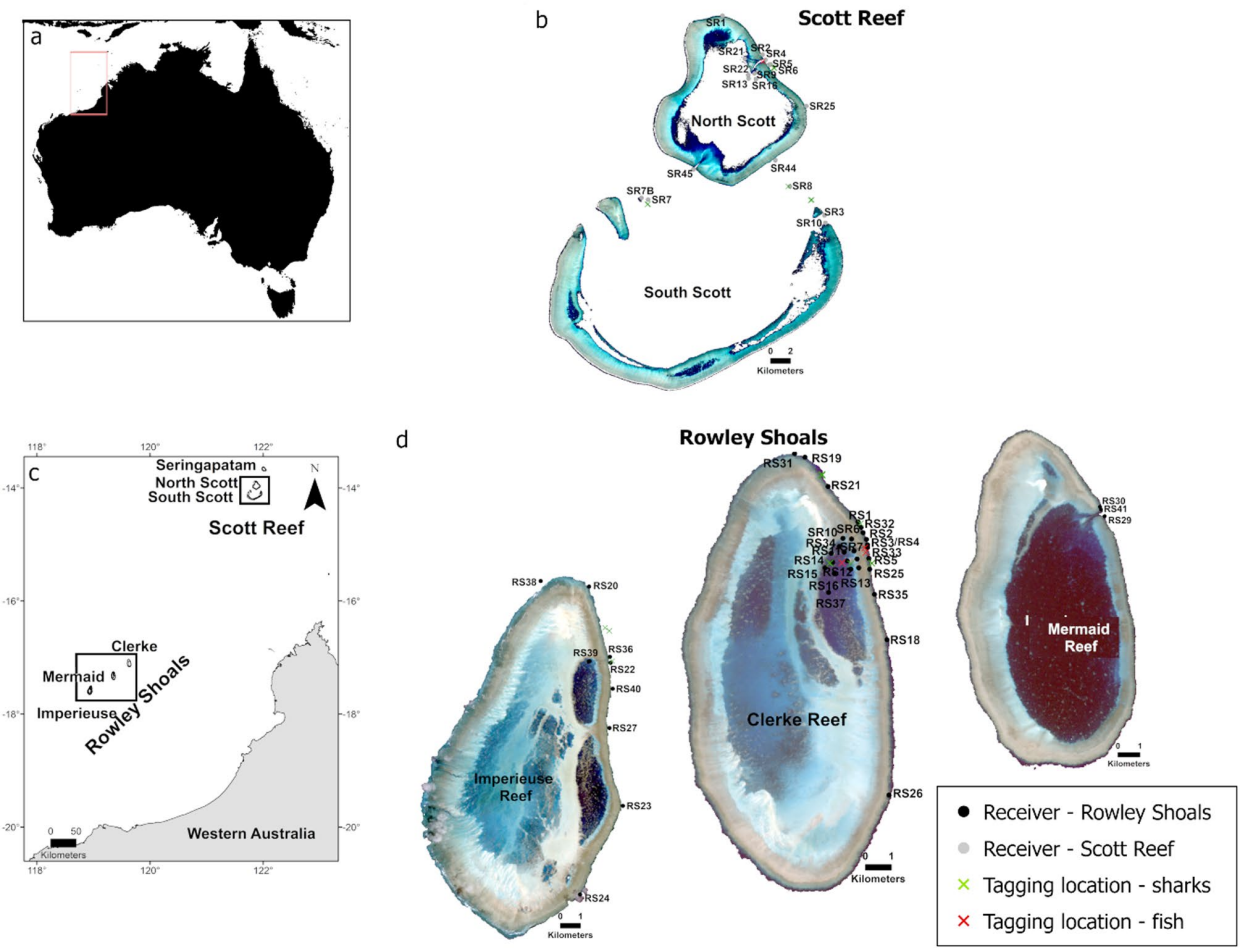
Map of Australia (**a**, top left) and Western Australia (insert; bottom left) indicating the location of Scott Reef and Rowley Shoals. Receiver locations at Scott Reef (**c**; top right) and Rowley Shoals (**d**; bottom right). Note that distance among atolls within Rowley Shoals atolls (~ 30 km) is not to scale

The study sites are the Rowley Shoals and Scott Reef, two coral reef systems each consisting of three atolls on the edge of the northwest continental shelf of Western Australia (Fig. 1). The empirical data spanned three spatial scales and encompassed the two reef systems. At local scales, we sampled within individual atolls (lagoons to forereef separated by metres to kilometres). We also made comparisons at regional scales, that is, among atolls within reef systems (separated by tens of kilometres), and between reef systems (up to 500 km between systems).

The three atolls that encompass the Rowley Shoals (Mermaid Reef, Clerke Reef and Imperieuse Reef; Fig. 1), are orientated in a north-easterly direction and separated by 30–40 km of deep (~ 350 m) water [20]. The Rowley Shoals have been protected as a Marine Park (Imperieuse and Clerke reefs) and a National Park (Mermaid Reef) for the last 30 years [20, 21]. The three atolls that make up Scott Reef are North Scott, South Scott and Seringapatam Reef (Fig. 1). These atolls are also located on the edge of the northwest continental shelf. Artisanal Indonesian fishing targeting reef sharks has occurred at Scott Reef since 1800s and is still permitted by the Australian Government [76, 95, 102].

### Acoustic tracking data

Existing acoustic tracking datasets for 63 grey reef sharks (*C. amblyrhynchos*), 10 silvertip sharks (*C. albimarginatus*) and 25 red bass (*L. bohar*) from Rowley Shoals and Scott Reef were accessed from the Australian Animal Acoustic Telemetry Database of the Integrated Marine Observing System (IMOS) housed in the Australian Ocean Data Network (AODN) portal https://portal.aodn.org.au/ [58]. These species were chosen to represent the top levels of the food web in coral reef ecosystems with large sharks representing top-order predators [50] and red bass as large mesopredators [4]. The data consist of detections of animals tagged with coded acoustic transmitters recorded on arrays of passive acoustic receivers deployed on the seabed. When a tagged animal is detected within a receiver listening range, the unique animal identifier, time and date, and potentially other variables depending on additional sensors present, are recorded [51, 53]. The animals were equipped with Innovasea V16 (sharks) and V13 (sharks and red bass) transmitters in 2007 [36], 2011, 2012 and 2015 (IMOS unpublished data). Tagging methods were described in detail in Field et al. [36]. Species were tagged at multiple shallow locations near the receiver array close to reef crest and within the reef lagoons (Fig. 1).

The Scott Reef receiver array, including 18 receiver stations (Fig. 1), was deployed as part of the IMOS national receiver network [58] along the north-eastern and southern sides of North Scott, inside the reef lagoon adjacent to the passage in the reef crest, and on the channel dividing North and South Scott between 2011 and 2016 (Fig. 1). Receivers in South Scott were deployed near the north-eastern edges of the reef crest. The receiver array at Rowley Shoals (41 stations, Fig. 1) was deployed in 2007 to match sites of a long-term monitoring dataset of coral and fish communities [36] on the eastern side of the three atolls. Range testing for receivers deployed at Imperieuse and Clerke reefs estimated a range of approximately 350 m [36], but we used a more conservative receiver range estimate of 300 m in our analysis as no range testing was conducted for Mermaid Reef and for the Scott Reef arrays (Fig. 1).

Tracking data in the Australian Animal Acoustic Telemetry Database undergo quality control checks; i.e., the validity of detections of each individual tag in the existing dataset is assessed based on a set of detection and movement metrics including distance between detections and to tagging location, velocity and detection distribution among others [58]. We filtered the tracking data to include detections with quality control type 1 (valid detection) and 2 (likely valid) [58] only and excluded individual animal tracks that were shorter than 7 days or those that were detected fewer than 50 times as these data would likely be heavily biased toward tagging sites due to animals leaving the array shortly after tagging. We also filtered the entire dataset to remove repeated animal detections below a 5-min resolution at each receiver to standardise the data and avoid false detections caused by collisions between transmissions of two transmitters [51]. We identified the periods of the day each detection was recorded using the R package *suncalc* [106]. The R package identifies time for sunset, sunrise, night start and end, etc., based on the local time (detection time stamp). We calculated the period of the day as: dawn, hours between the end of night and end of the golden hour; day, hours between end of golden hours and sunset; dusk, between sunset and start of night; night, hours between start and end of night.

### Network analysis

Acoustic detection data were used to create movement-based connectivity matrices, where each element represented a pairwise movement event (i.e., edges; consecutive detections of a unique animal tag from one receiver to another receiver). This data structure captures directionality of movements from a receiver (unique row) to all other destination receivers (columns) and the strength of movement represented by the magnitude (large values for repetitive movements between receiver pairs). This movement matrix was used to produce directed and weighted networks to represent the spatio-temporal dynamics of each species. The number of directed edges, defined as the number of animal movement events between pairs of receiver stations, and the number of successive detections at the same station (self-loops), were used in the network analysis. The time between detections was also used as weight for edges by log-transforming the inverse of the time between successive detections and then summing all values from all individual edges between a pair of receiver stations (or the same receiver) to represent the weight of the specific edge. This was done so that edges representing movements with shorter time between detections had greater strength. We developed networks for each species at each reef system (Scott Reef and Rowley Shoals) using the full dataset for the species, for each time of the day (dawn, day, dusk and night), and each sex (sharks only). We also created individual networks for each individual shark or fish to characterise movement, determine core use areas and connectivity across the entire coral reef system. Finally, we used all individual movement networks to quantify habitat preferences and their influence on movement patterns. Only those individual-level networks consisting of movements across three or more nodes (receivers) were included in the analysis.

We calculated multiple metrics to describe properties of networks, nodes and edges using the software R [89] and packages *igraph* [18] and *sna* [15]. For each network we calculated metrics to provide broad-scale diagnostics for the extent of movement for each species and individual. The network order (the number of nodes) and size (the number of edges) were recorded as simple proxies of total movement. We also recorded the number of components in each network, defined as unique clusters of connected nodes that are separated from other parts of the network. The number of components is an indication of fragmentation in the network, and is used to identify movement barriers [11].

Centrality measures were used to identify important core use areas and corridors in each network. Degree centrality (*d*) is a local centrality measure quantifying the number of edges adjoining the node, including all in- and out-going edges for each node [91, 108] indicating how locally connected a node is to its neighbours. High degree centrality nodes (i.e., hubs) are often important for maintaining local-scale movement and cohesion in the network [91, 108]. Edge betweenness centrality (*b*) was calculated for all networks, quantifying the frequency of shortest paths in a network that use each movement link. Edge betweenness identifies links and pathways that are high-use and, therefore, promote system-wide connectivity across the network [61]. Common pathways within each reef system were identified by comparing values of edge betweenness for edges shared by two or more species. We then identified the shared edges with highest betweenness values at each reef system and considered those as common important pathways.

### Habitat preference

Since benthic characteristics play a significant role in driving distribution patterns in many marine species [38, 88, 101], we assessed the influence of habitat type in the core use areas and the associated connectivity for each species at each reef system. Values for node degree centrality from individual networks of each species (grey reef sharks, silvertip sharks and red bass) at Rowley Shoals and Scott Reef were used as a measure of core area. Only individual networks with three or more nodes were included in the analysis.

Information on the benthic habitat at each receiver station was extracted from existing spatial habitat and depth models for Scott Reef [56, 90] and the Rowley Shoals [46] for the area within the receiver listening range (300 m radius). The categories from the models were combined into broader classes which are expected to drive the movement and behaviour of coral reef fishes [9, 44]. The models were derived from combining Sentinel 2 satellite, Lidar and multibeam remote sensing datasets [46, 56, 90]. Each habitat model conformed to a consistent and common CATAMI (Collaborative and Annotation Tools for Analysis of Marine Imagery and Video) habitat classification scheme [3]. From these spatial habitat models, we calculated the proportion of each habitat category (Table S1) in the surrounding area of the 300 m radius (proxy to receiver range) around each receiver station. In addition, we extracted depth, distance to nearest reef crest (dist.reef) and distance to main passage in the reef crest (dist.channel) for each receiver station (node) using the Euclidean distance tool in ArcGIS 10.4 [32].

Gradient boosted models (GBMs) were then used to analyse the relationship between node metric (degree) and habitat variables (Tables S1 and S2) for each species at each reef system. The explanatory variables included habitat categories extracted from the habitat model (Table S1), but also depth at the receiver location, distance from each node/receiver to the reef crest and the distance from each node/receiver to the passage in the reef (Table S2). We also included the sex (for sharks) and time of day (categorical dawn/day/dusk/night) as variables in the model. All analyses were conducted in R using the packages *gbm* [45] and *caret* [68]. Gradient boosting is a machine learning algorithm that sequentially adds new trees to train initial weak and shallow trees. At each iteration, new trees are built from a random subsample of the data, and the model focuses on incrementally correcting predictions based on the error learned by earlier trees to minimise loss of predictive performance or deviance of the model [26, 86]. We split each dataset into a training (75% of the available data) and a testing (remaining 25% of data) subset before each analysis. Model settings (interaction depth, bagging fraction, shrinkage rate and training fraction) were specifically optimised for each response variable by performing a grid tuning routine over every possible combination of the model settings parameters. The final model setting was selected by the lowest relative mean squared error and this model was then used to identify the most important explanatory variables. We used the Spearman correlation coefficient to assess accuracy of the model by comparing modelled results against the testing subset.

## Results

### Acoustic tracking

The 59 receiver stations (nodes) across the two reef systems recorded 938 unique movements (edges) from 78 individuals across all three species. Tracking data for 54 grey reef sharks (19 males, 33 females and two with unknown sex), 7 silvertip sharks (five males and two females) and 17 red bass (unknown sex) were included in the analysis (Table 1). Most grey reef sharks (n = 44) and red bass (n = 13) were tracked in the Rowley Shoals, whereas tracking data from silvertip sharks was only available for Scott Reef (Table 1).

**Table 1:** Acoustic detection data downloaded from the Australian Animal Acoustic Telemetry Database as part of the Integrated Marine Observing System from acoustically tagged grey reef sharks, silvertip sharks and red bass at Rowley Shoals and Scott Reef indicating the tag deployment year, total length of tagged animals, number of detections and duration in days that each tagged animal was detected in the receiver array, and number of tagged individuals of each sex. Total length is given as mean ± SD and number of detections and duration as median and range. M = male, F = female and U = unknown sex

	Deployment	Total length (cm)	Number of Detections	Duration (days)	Number of individuals			
					M	F	U	Total
**Grey reef shark**			6647 (74–45,121)	429 (20–1422)	19	33	2	54
Rowley Shoals	2007, 2011,2012	146.1 ± 19.5	5413 (74–39,044)	458 (20–1415)	16	26	2	44
Scott Reef	2011	131.1 ± 30.7	12,077 (101–45121)	639 (212–1422)	3	7		10
**Silvertip shark**	2011	117.7 ± 20.1	836 (73–1746)	617 (68–1029)	5	2		7
**Red bass**			1257 (56–7885)	309 (46–499)				17
Rowley Shoals	2015	58.7 ± 4.8	1547 (56–7885)	314 (46–499)				13
Scott Reef	2015	48.8 ± 16.0	131 (84–661)	294 (68–488)				4

### Network analysis

#### Scott reef

##### Grey reef sharks

**Fig. 2:**
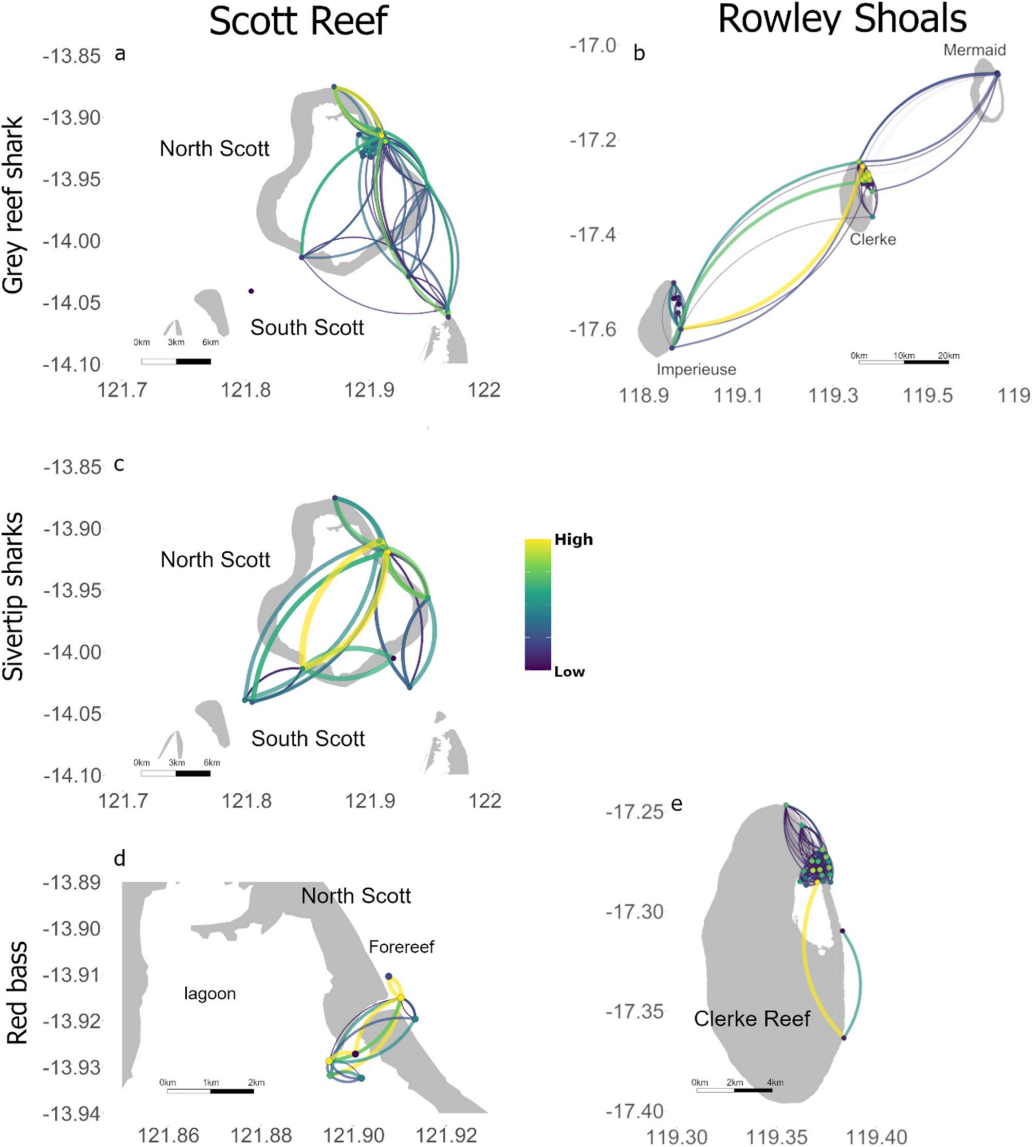
General networks for grey reef sharks (**a**, **b**), silvertip sharks (**c**) and red bass (**d**–**e**) at Scott Reef (**a**, **c** and **d**) and Rowley Shoals (**b** and **e**). Note that panels for red bass were zoomed to North Scott near the passage in the reef (**b**) and to Clerke Reef only (**b**) due to their more restricted movement extent in relation to sharks. The colour scale indicates values of degree for each node and betweenness for each edge, however they were scaled as high-low within each network to aid visualisation

At Scott Reef, the general grey reef shark network consisted of 17 receiver stations or nodes, and 107 weighted directional movements pathways or edges (Table 2, Fig. 2a). The grey reef shark network had two separate components, one that included both North Scott and South Scott with many directional movements, and a second represented by an individual node on the northwest side of the channel between the reefs detecting only one shark and not linked by movement paths to other nodes in the network (Fig. 2a). The area of highest use, represented by nodes with highest number of connections (*d* = degree), was located in front of (outside the reef) the northeast passage (Fig. 2a) (*d* = 25 and 19; Table S2). Most important pathways (higher value of betweenness; *b*) linked the high use areas near the passage with the northern extent of North Scott (*b* = 14 to 21, Table S3), but also to South Scott (*b* = 13 to 15) (Fig. 2a, Tables S2 and S3). This was their core use area during all times of the day (*d* = 10,19, 12 and 14 during dawn, day, dusk and night, respectively; Fig. S1, Table S2) with most important movement pathways representing restricted movement within North Scott, including movement linking the lagoon and offshore areas through the northeast passage (Fig. S1a, d, g, j). Overall, the day, dusk and night networks for the grey reef shark at Scott Reef had a similar size (number of nodes) and six components, but grey reef sharks displayed a larger number of directional movements (size) during the day and the lowest number of directional movements, and smallest network, at dawn (Table 2).

**Table 2:** Calculated network metrics for grey reef sharks, silvertip sharks and red bass (full), for each sex, and for each period of the day (dawn, day, dusk and night) at Scott Reef and Rowley Shoals. Order indicates number of receivers with detections from tagged animals in each network, size is the number of directional movements between receivers, and components indicate the number of components or subgraphs in each network

Network	Scott reef			Rowley Shoals		
	Order	Size	Components	Order	Size	Components
Grey reef shark						
*Full*	17	107	2	38	513	3
Dawn	8	22	3	33	249	7
Day	17	64	6	37	396	5
Dusk	13	32	6	33	247	9
Night	17	46	6	38	347	7
*Female*	16	82	1	37	450	3
Dawn	7	18	2	31	193	6
Day	15	45	5	36	328	4
Dusk	10	22	5	31	167	8
Night	13	32	6	36	281	6
*Male*	15	74	2	31	437	3
Dawn	6	17	3	27	196	4
Day	13	45	4	30	348	5
Dusk	10	28	4	26	220	5
Night	13	35	3	28	293	4
Silvertip shark						
*Full*	11	46	1			
Dawn	5	9	3			
Day	9	24	4			
Dusk	7	14	4			
Night	8	18	5			
*Female*	11	43	1			
Dawn	4	8	2			
Day	8	23	4			
Dusk	6	13	3			
Night	8	18	5			
*Male*	6	12	1			
Dawn	1	1	1			
Day	5	5	3			
Dusk	2	2	2			
Night	2	2	2			
Red bass						
*Full*	7	24	1	22	248	1
Dawn	3	3	3	19	70	1
Day	6	14	2	18	145	1
Dusk	1	1	1	17	41	4
Night	6	9	3	18	91	1

**Fig. 3:**
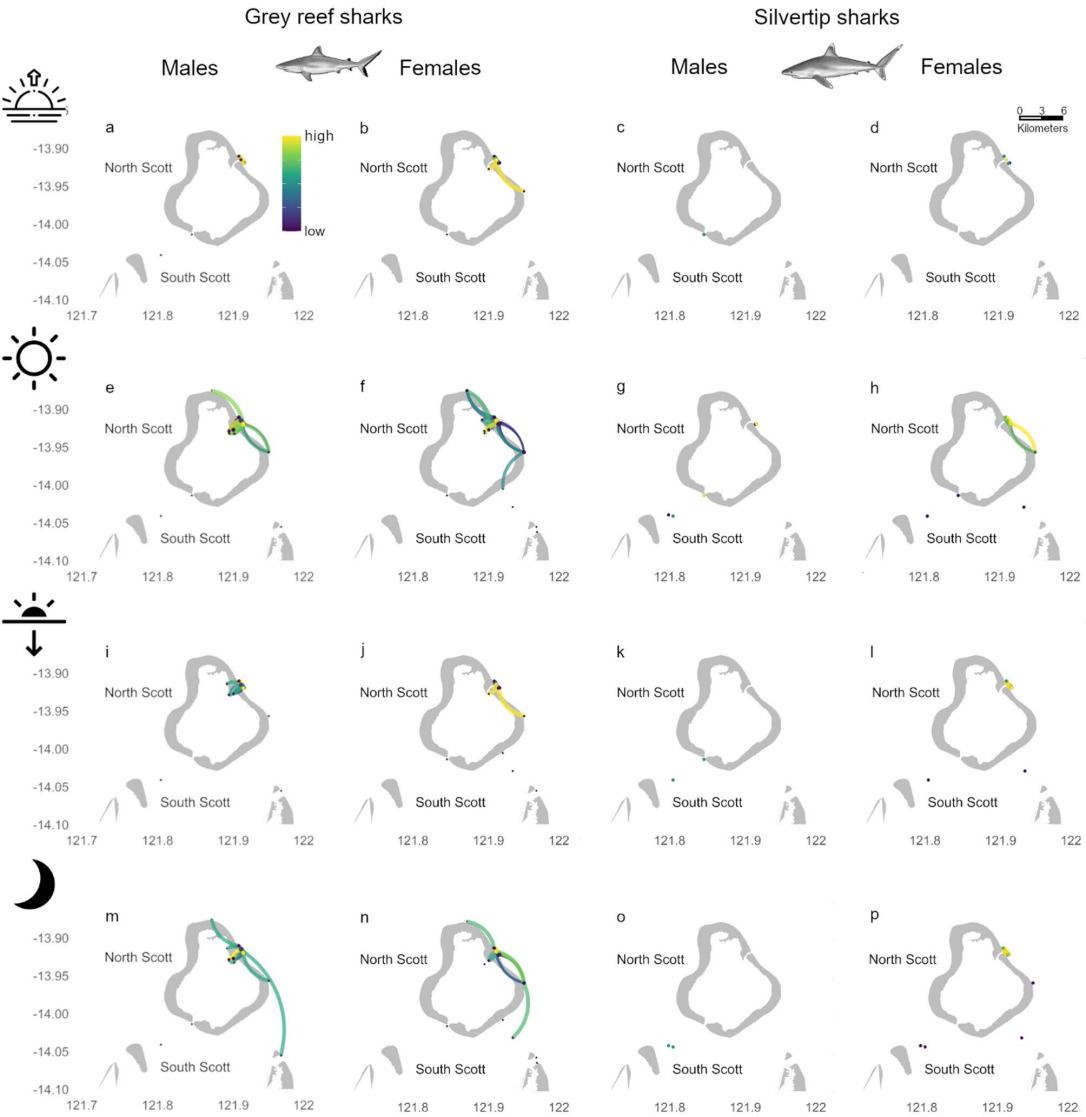
Networks for grey reef sharks (**a**, **b**, **e**, **f**, **i**, **j**, **m**, **n**) and silvertip sharks (**c**, **d**, **g**, **h**, **k**, **l**, **o**, **p**) at Scott Reef. Panels indicate males (**a**, **c**, **e**, **g**, **I**, **k**, **m**, **o**) and females (**b**, **d**, **f**, **h**, **j**, **l**, **n**, **p**), and times of the day: dawn (**a**–**d**), day (**e**–**h**), dusk (**i**–**l**) and night (**m**–**p**). The colour scale indicates values of degree for each node and betweenness for each edge, however they were scaled as high-low within each network to aid visualisation, directionality of edges is represented in a clockwise movement

Networks differed by sex for tagged grey reef sharks (Fig. 3a–d). The network for female grey reef sharks was larger with more nodes and a larger number of directional movements between nodes. The male network had two components while the female had only one (Table 2, Fig. 3a–d). Both sexes of grey reef sharks showed consistent high use of the area in front of the northeast passage, independent of period of the day (*d* = 10, 14, 9 and 12 for females during dawn, day, dusk and night, respectively; and *d* = 8, 18, 12 and 12 for males during dawn, day, dusk and night, respectively), however, high use movement paths and size of networks changed for each period of the day (Fig. 3a–d). Networks were larger during the day and night when compared to dawn and dusk for both sexes (Table 2). For males, movement paths were limited to the area just in front of the northeast passage at dawn, with sharks were going in and out of the lagoon during the day and moving further along North Scott (Fig. 3a, e). At dusk, movement pathways were restricted to the northeast passage, whereas at night male sharks showed the greatest extent of movement linking North and South Scott (Fig. 3i, m). Female sharks remained near the northeast passage during crepuscular periods (dawn and dusk). During the day, female sharks moved further along the forereef of North Scott and showed greater use of the lagoon (Fig. 3f). At night, similar to male sharks, movement pathways of female sharks linked North and South Scott (Fig. 3n).

##### Silvertip sharks

The general network for silvertip sharks was composed of 11 nodes and 46 directional movements (Table 2). Use of the area in front of the northeast passage in the reef was high (*d* = 14, Table S2), but there was no use of the lagoon (Fig. 2c). This pattern was consistent at all times of the day (*d* = 6, 10, 6 and 8 for dawn, day, dusk and night, respectively; Figure S1d, e, h, k). The most important pathway for the movement network was also near the northeast passage with more extensive movements during the day (Fig. S1e). Networks for each time of the day had multiple components with one or more individual nodes left isolated (Fig. S1d, e, h, k). The overall scale of movements (network order and size) was similar for all times of the day except at dawn when the network was smaller and with less directed movements (Table 2, Fig. S1d, e, h, k).

Female silvertip sharks showed a greater level of movement (number of directed movements and nodes) than males in general (Table 2, Fig. 3c, d, g, h, k, l, o, p). However, only two female silvertip sharks were included in the analyses (Table 1). Females showed consistent high use of the area in front of the northeast passage at North Scott (*d* = 6, 10, 6 and 8 for dawn, day, dusk and night networks, respectively; Fig. 3d, h, l, p) with movements pathways mostly restricted to that area during all periods of the day (Fig. 3d, h, l and p). Whereas males were detected by individual receivers not linked to others near the southwest passage and in the channel between North and South Scott (Fig. 3c, k, o), except during the day when they also used the areas in front of the northeast passage (Fig. 3g).

##### Red bass

The red bass general network consisted of 7 nodes and 24 directional movements (Table 2) and was restricted to the area near the northeast passage (Fig. 2d). Red bass displayed highest use of discrete areas at both ends (inside and outside) of the northeast passage (*d* = 11, Table S2) and movements across the northeast passage represented the most important pathway (*b* = 6; Fig. 2d, Table S3). The use of the area near the northeast passage was consistent throughout the day (d = 2, 6, 2 and 6 for dawn, day, dusk and night, respectively), however the extent of movement varied (Fig. S1c, f, i, l). At dawn and dusk, movements were restricted to individual receivers not linked with others. At dawn, red bass was detected both inside and outside the lagoon (Fig. S1c) but they were detected outside the lagoon only at dusk (Fig. S1i). During the day, movements linked nearby receivers inside and outside the lagoon with no movements through the passage (Fig. S1f), whereas at night, red bass movements linked the areas inside and outside the lagoon though the northeast passage resulting in a larger network with multiple components (Fig. S1l, Table 2).

#### Rowley shoals

##### Grey reef shark

At the Rowley Shoals, movements of grey reef sharks linked all three atolls encompassing 38 nodes and 513 directional movements (Fig. 2b, Table 2). Shark movements created links between Clerke and Imperieuse reefs and Clerke and Mermaid reefs, but no direct movements were observed between Mermaid and Imperieuse (Fig. 2b). The network had 3 components that included nodes from multiple atolls (Table 2, Fig. 2b). The Rowley Shoals general network for grey reef sharks showed Clerke Reef to have the highest use (*d* = 33 to 47; Fig. 2b, Table S2), with movements between Clerke and Imperieuse showing the highest importance (*b* = 110). This high use of Clerke Reef was also observed during different times of the day, however, large scale movement between distant reefs was not observed when considering each time of the day separately (Fig. S2). At Clerke Reef, areas of high use included the lagoon at dawn and dusk, and areas outside the lagoon during the day and an at night (Fig. S3). Additionally, grey reef sharks moved larger distances resulting in larger and more connected networks (with lower number of components), during the day and at night (Table 2).

**Fig. 4:**
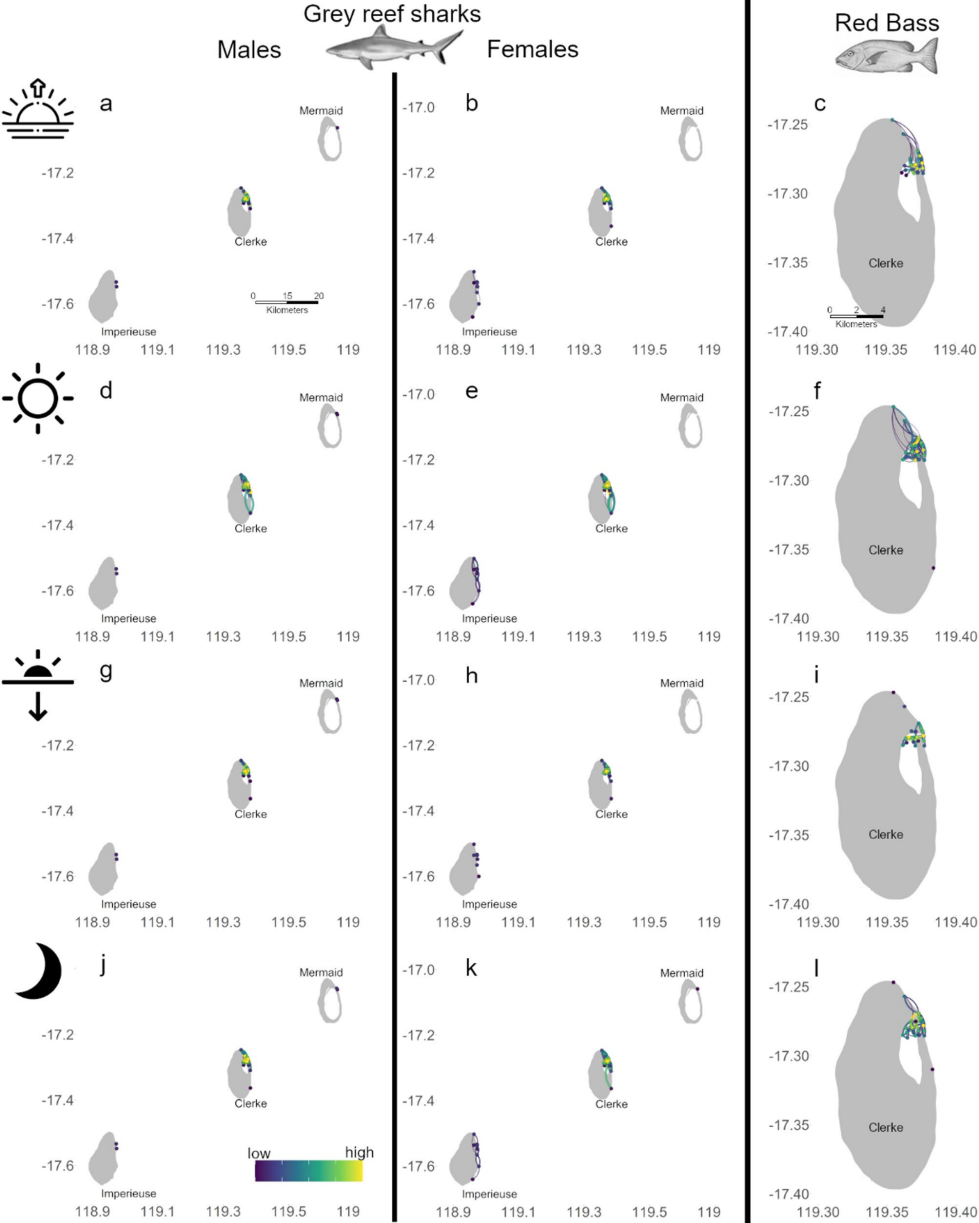
Networks for grey reef sharks (**a**, **b**, **d**, **e**, **g**, **h**, **j**, **k**) and red bass (**c**, **f**, **I**, **l**) at Rowley Shoals. Panels indicate male shark networks (**a**, **d**, **g**, **j**) and female shark networks (**b**, **e**, **h**, **k**), and red bass during dawn (**a**–**c**), day (**d**–**f**), dusk (**g**–**i**) and night (**j**–**l**). The colour scale indicates values of degree for each node and betweenness for each edge, however, they were scaled as high-low within each network to aid visualisation, directionality of edges is represented in a clockwise movement

Female grey reef sharks in the Rowley Shoals moved through a larger area and connected all three distant atolls, with large-scale movements linking Clerke and Mermaid reefs, and Clerke and Imperieuse reefs. Male grey reef sharks also performed large-scale movements, however, these happened between Clerke and Mermaid reefs only, with sharks tagged at Imperieuse Reef not being detected in other atolls (Fig. S4). Clerke Reef displayed highest used areas and most important pathways for both sexes (Figs. 4 and S4) and during all periods of the day (Fig. 4). During the day, both males and females displayed larger networks that were more connected (and fewer components) than other periods of the day (Table 2). Females displayed high use of areas both inside and outside the lagoon at Clerke Reef (Fig. S3f) and males showed high use of the area adjacent to the passage outside the lagoon at Clerke Reef (Fig. S3e). At Clerke Reef, both sexes also showed higher use of the lagoon at dawn and dusk (Fig. 4a-b, g-h; S3b, c, h, i).

##### Red bass

The red bass network consisted of 22 nodes and 248 directional movement concentrated at Clerke Reef only (Fig. 2e). Overall, red bass showed higher use of the lagoon habitat (*d* = 34; Table S2) with the most important pathway linking the lagoon to southern areas of the forereef (*b* = 40; Table S3). However, most movement paths were concentrated within the lagoon and to/from the northern section of the reef (Fig. 2e). The network order was similar throughout the day; however, the number of directed movements was greater during the day and smallest at dusk (Table 2; Fig. 4c, f, I, l), although during dusk the network had the highest number of components (Table 2). The most important area used was located just in front of the passage outside the lagoon (d = 13, 10, 17 for dawn, dusk and night, respectively; Fig. 4c, i, l) except during the day when the most important area was located near the passage but inside the lagoon (d = 25; Fig. 4f). The most important pathways represented movements within the lagoon at dawn and dusk (Fig. 4c,i), and movements linking the inside and outside of the lagoon during the day and at night (Fig. 4f,l).

### Common pathways

**Fig. 5:**
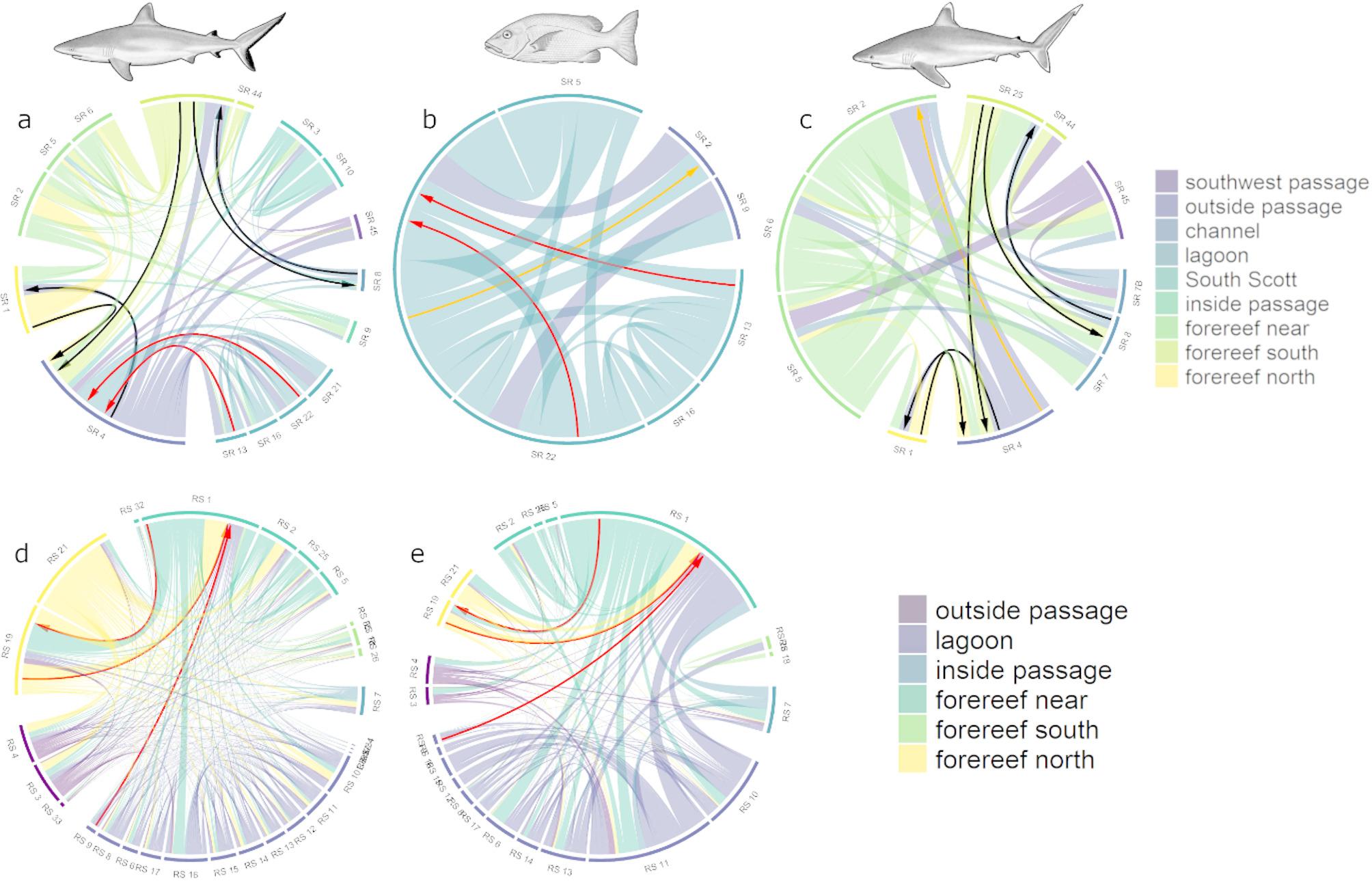
Edge betweenness connectivity plot for grey reef sharks (**a**, **d**), red bass (**b**, **e**) and silvertip sharks (**c**) for Scott Reef (**a**–**c**) and Rowley Shoals (**d**–**e**). Size of each link indicates values of betweenness (thicker lines are higher values). Common high-use corridors are identified as edges with high betweenness shared by two or more species as arrows: black are common corridors between grey reef sharks and silvertip sharks, red are common corridors between red bass and grey reef sharks and orange are between silvertip sharks and red bass. The axis indicates the receiver IDs (see Fig. 1 for their locations) and the colours indicate they general location within the atolls (*inside passage* = receivers in front of the passage in the reef crest in the lagoon, *outside passage* = receivers just outside the passage; *forereef near* = receivers in the forereef near the passage in the reef, *forereef south* = receivers in the southern extent of the atoll; *forereef north* = receivers in the northern extent of the atoll

At Scott Reef, there were common important pathways (directional movement paths used by multiple species, with high betweenness values) among all three species (Fig. 5a). The common pathway with highset use between grey reef sharks and silvertip sharks occurred along the reef (outside the lagoon) at North Scott (SR1 <—> SR4; SR25- > SR4; Fig. 5a,c), and also to/from the channel between the atolls (SR25 <—> SR8; Fig. 5a,c). Grey reef sharks and red bass shared multiple directional movement paths but only two displayed high betweenness for both species (Fig. 5a, b). These represented movement within the lagoon (SR13- > SR 4; Fig. 5a, b) and movement through the northeast passage (SR22- > SR4; Fig. 5a, b). Red bass and silvertip sharks shared one common pathway only (Fig. 5b, c) represented by movements just outside the northeast passage at North Scott (SR4- > SR2; Fig. 5b, c).

At Rowley Shoals, the most important common pathways shared by grey reef sharks and red bass occurred along the forereef at the northern section of Clerke Reef (SR1 <—> SR19; Fig. 5d-e) and movements in and out of the lagoon (RS9 <—> RS1; Fig. 5d, e).

### Habitat preferences

Habitat preferences for each species were assessed using individual networks for 54 grey reef sharks (44 at Rowley Shoals and 10 at Scott Reef), 6 silvertip sharks (Scott Reef only), and 13 red bass (Rowley Shoals only) (Table 1).

**Fig. 6:**
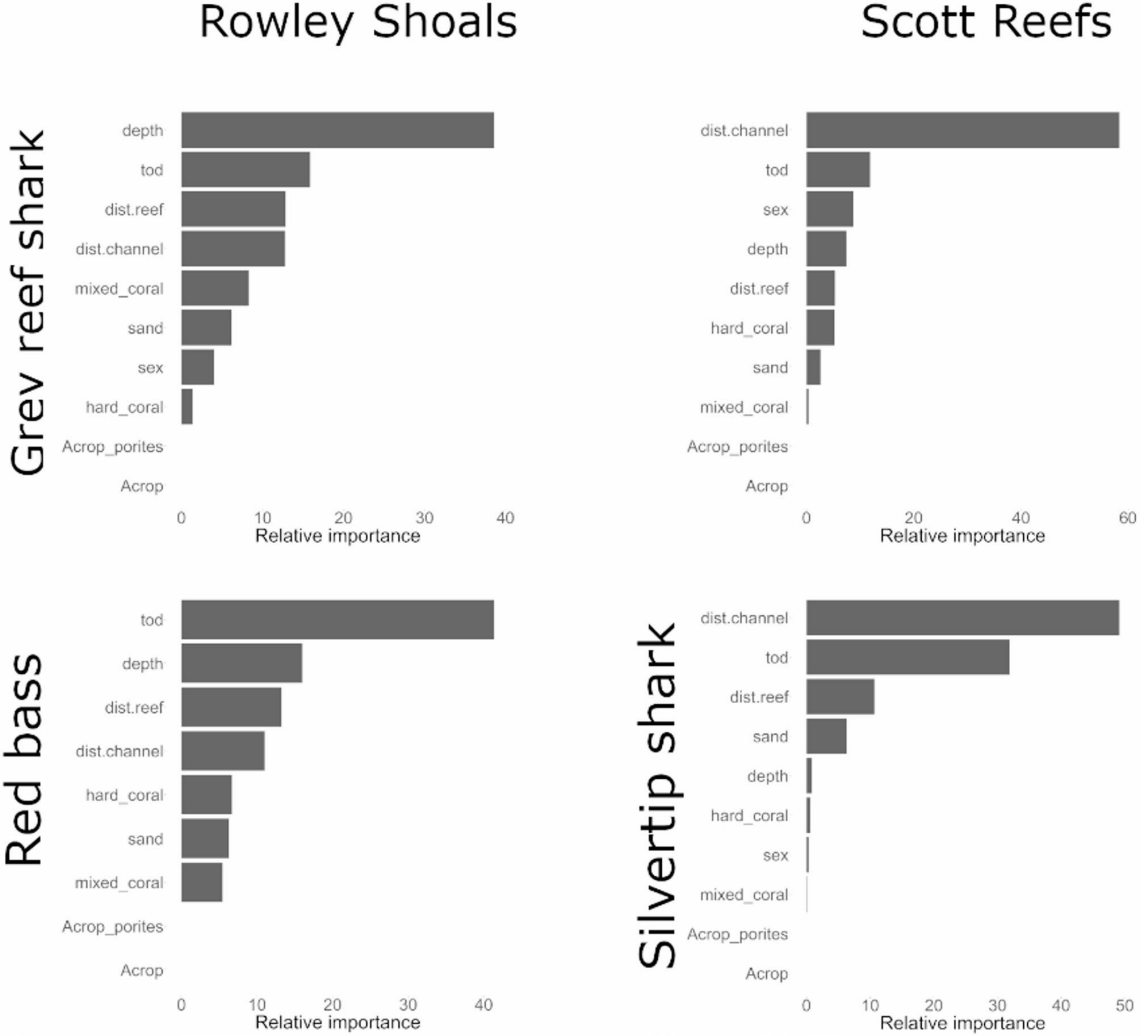
Relative importance from gradient boosted models for covariates explaining centrality measures (degree) of all individual networks of grey reef sharks at Rowley Shoals and at Scott Reef (top 2 panels), red bass at Rowley Shoals (bottom left panel) and silvertip sharks at Scott Reef (bottom right panel). Details of each explanatory variables are summarised in Tables S1 and S2

Gradient boosted models (GBM) identified the most important predictors for core use areas for grey reef sharks at Scott Reef and Rowley Shoals, silvertip sharks at Scott Reef and red bass at Rowley Shoals. We were not able to fit GBMs for red bass at Scott Reef due to an insufficient number of individual networks with more than three nodes for this species. Distance to the main passage in the reef crest was identified as the top predictor for sharks at Scott Reef (Figs. 6, S5–S8). Time of the day (*tod*) was the top predictor for red bass, and the second most important predictor for sharks at Rowley Shoals and Scott Reef (Fig. 6). Depth was the top predictor for grey reef sharks and the second most important for red bass at Rowley Shoals (Figs. 6, S5–S8). All GBMs for grey reef sharks had very good model fit with R^2^ values above 0.7, and GBMs had moderate fit (0.4) for silvertip sharks and for red bass.

## Discussion

We show how animal tracking, network analyses and machine learning can provide a unique understanding of the complexities of animal movements driving seascape connectivity within and between remote coral reef ecosystems over local and regional spatial scales. In general, core use areas were represented by low complexity habitats (dominated by sand) near the passage in the reef crest. However, patterns of movement and core habitat use varied among species, time of day and sex. Overall, female sharks (grey reef and silvertip) had larger networks representing greater movement extent than males. Red bass movements were more restricted than those of sharks with a preference for lagoon habitat and areas near passages in the reef crest. Silvertip sharks were only tagged at Scott Reef and were not detected inside the lagoon. Grey reef shark movements resulted in greater connectivity within each reef system than silvertip sharks and red bass, linking different atolls within a system. The preference for shallow water near the reef crest suggests that protection of atoll lagoons at Rowley Shoals is sufficient to protect red bass. However, the large networks and extensive movements of grey reef sharks (> 30 km) suggest that current marine protected areas do not protect the seascape connectivity these sharks require within and among coral reef systems in Northwest Australia. The patterns in connectivity within atolls (between lagoon and forereef, and along forereef) and between atolls within a reef system highlight both the importance and the challenges of integrating movement patterns in a spatial planning framework, particularly for highly mobile species such as large predatory fish and megafauna.

The movement networks of grey reef sharks in both reef systems covered a greater distance than the other two species, consisting of a mix of local hubs of neighbour nodes with occasional movements among distant (> 30 km) atolls within a reef system. Long distance movements by grey reef sharks, although not common, have been recorded for the species elsewhere [6, 12, 55, 118], with acoustically tagged sharks undertaking migrations of up to 340 km along the barrier reef surrounding Nouméa in New Caledonia [12] and satellite tagged sharks moving over 900 km in pelagic waters surrounding Palmyra Atoll [118]. However, these studies showed the opposite pattern seen in our networks, with males displaying more extensive movements than females. Previous acoustic tracking of grey sharks have been conducted on fairly connected or contiguous reef [12, 55], with habitat continuity being hypothesised to be a determinant factor of large distance movements for the species [12, 55]. We show that in oceanic reef systems, habitat continuity is not a limiting factor for grey reef sharks and that occasional long-distance movements are relatively common. Large scale movements of grey reef sharks have been attributed to reproduction [55] and to maximize foraging opportunities [29]. Given that the mean total length of the grey reef sharks tagged at Rowley Shoals (146.1 ± 19.5 cm) indicate they were mostly mature individuals [117], it is possible that extensive movements of females are linked to reproduction such as partial migration to pupping and nursery areas [30, 100]. However, further investigation is needed to understand the drivers for such large-scale movements.

The network for silvertip sharks was smaller and denser than the grey reef shark network at Scott Reef indicating more local-scale movements. The lack of long-distant movements observed here contrasts to that seen in previous studies, which reported that silvertip sharks moved greater distances and had more complex movements than other reef sharks [17, 28, 31, 63]. A few factors might have resulted in the movement patterns we describe for Scott Reef. Only seven silvertip sharks were included in the analysis (cf. 54 grey reef sharks) and they were mainly subadults (mean length of tagged silvertip sharks = 117.7 ± 20.1 cm; length at maturity = 174.7 cm and 208.9 cm for males and females, respectively [99]). Another possibility is that the receiver array failed to include preferred habitat by silvertip sharks such as reef slopes [69] (Fig. S1) which were not monitored by our acoustic array. Hence, it is possible that a spatial segregation exists between the top-order predators in this oceanic system with grey reef sharks showing preference to reef-associated habitat, including the reef lagoon, with silvertip sharks being more common in the pelagic zone with minor use of near reef habitats where receivers were deployed. As a result, grey reef sharks might effectively act as apex predator in the reef lagoon in the absence of larger shark species [50], whereas further competition, and predation risk, exist in the reef front and slope habitats.

All species showed larger networks and more extensive movements during the day and at night, with some variation of the areas of high use during different period of the day. More extensive movements have been reported during the crepuscular periods for several predatory shark species [19, 40, 47] and Lutjanid fishes are considered a nocturnally actively species [84]. However, we observed a different pattern. It is possible that during crepuscular period sharks are leaving the areas near the reef and targeting prey in the pelagic habitat further from the reef crest, which were not monitored by our receivers and thus presenting a false sense of reduced movement during periods of dawn and dusk. For example, in Palau, grey reef sharks use greater depths during crepuscular hours and at night [113]. The high level of individual variability in movement and habitat preferences of these predators (temporal and spatial variability in networks) may suggest our sample size was not sufficient to identify the main drivers of their diurnal movement patterns. Other factors influencing movement such as tides, lunar phase and thermal preferences and regulation [103, 113] are also likely influencing the patterns that we show here.

Both grey reef and silvertip sharks also displayed differences in connectivity and movement patterns between males and females, with females exhibiting larger networks and more connections between nodes. Although high-use areas were mostly consistent between sexes, the most important pathways, and thus the connectivity patterns, differed. These results suggest the movement of females create increased connectivity and that males and females create different connectivity pathways at finer spatial scales (within an atolls or reef). Intra-specific sex-specific movements and spatial segregation are common across many shark species [62, 81, 85, 115], and often relate to different environmental and energy requirements for reproduction [98, 105], growth [77, 81], and as females avoid harassment from males [25]. Although our results do not seem to show clear patterns of spatial segregation, the differences in movement and connectivity patterns suggest potential sex-specific differential space use within Rowley Shoals and Scott Reef. Spatial segregation can have severe implications for conservation and management as it might increase the exposure of a portion of the population to threats such as fishing [66, 82, 101, 115].

Our network and centrality metrics suggest that red bass displayed higher site fidelity and more restricted movement than reef sharks. The smaller network for the red bass compared to sharks is likely a combination of their high site fidelity and the configuration of receiver arrays, with receivers within the lagoon and near passages closer to each other than receivers along the forereef (Fig. S1). Red bass diurnal movement patterns were similar in both reef systems, with larger networks and movement during the day and at night than during crepuscular periods. Limited information is available on the movement patterns of red bass and most observations relate to spawning aggregations [22, 96]. It is possible that these predators are moving away from areas monitored by our array due to increased mobility during crepuscular hours when prey leave their hiding spots, although we cannot confirm this without tracking the movement of potential prey. However, our results highlighted their preference for shallow waters near passages in the reef crest and increased movements linking the lagoon with the forereef during the day and night, creating localised but strong links with the forereef, which can be used to inform spatial planning for the species.

Unlike kernel density methods, a combination of network methods are necessary to define core areas using metrics of shark movement networks [70]. Network analysis, nevertheless, can provide a more complete and system-wide picture of movement patterns, space use and habitat partitioning within dynamic and complex systems [31, 52, 69, 70, 80]. The preference for areas near the passage in the reef crest (albeit also near some of the tagging sites) was consistent between species and reef systems. The high use of passages was further supported by the identification of multispecies common pathways for grey reef sharks and red bass. However, it is also possible that some of this habitat preference pattern has been influenced by the proximity to tagging sites, with many individuals, particularly red bass, tagged in shallow areas relatively close to reef passages (Fig. S1).

All species preferred habitats with low structural complexity (sand cover) which are the dominant habitats between reef patches and areas of higher coral cover. These habitats are often referred to as edge habitats, and commonly shown to be preferred by carnivorous fishes [97]. In contrast, on the Great Barrier Reef, occurrence of grey reef and silvertip sharks in Baited Remote Video (BRUV) deployments has been linked to proximity to reef and hard coral cover [27]. However, the bait plume from BRUVS attracts animals from surrounding areas [39, 48] hampering direct comparison between methods, thus animal tagging may provide a more realistic representation of habitat use. This preference could have resulted from foraging strategies [93], with animals patrolling these edge habitats with minimal protection from the reef for prey. Although the preference for sandy habitats is likely to be influenced by the choice of receiver deployment locations as the array was not able to cover all potential habitats available to highly mobile species, the high number detections during the deployment period (Table 1) suggests tagged animals were continuously moving though the area monitored by receiver arrays.

Marine protected areas (MPAs) can be an important spatial management tool for the protection of mobile predators when properly enforced [17, 102, 104, 118]. However, their efficacy is greatly affected by the size of the protected areas and scale of movements of mobile species [24, 27, 31, 54, 69, 101]. The current management plan for Rowley Shoals, with fishing prohibited within lagoons and at Mermaid Reef ([20, 21]), might offer a high level of protection for red bass movement and connectivity. However, movement of grey reef sharks across oceanic waters between Rowley Shoals’ atolls will still expose them to a range of threats [34, 35], albeit monitoring against illegal fishing is strictly enforced in Australia [102]. Nevertheless, the marine park management plan would benefit from considering the seascape connectivity created by extensive movements of grey reef sharks within Rowley Shoals. Despite the lower sample size and more restricted coverage of the receiver array at Scott Reef, the high connectivity between North and South Scott created by movements of grey reef and silvertip sharks also suggests these sharks might have greater exposure to fishing pressure from artisanal Indonesian fishers than red bass at that locality.

There is growing evidence to support the need for effective management strategies to account for ecological connectivity in marine ecosystems [10, 31, 73, 107, 108]. Our findings demonstrate that movement of marine predators creates connectivity among different habitats within a coral reef atoll but also across distant areas within a reef system. Our analyses suggest that existing marine protected areas at each atoll of the Rowley Shoals may be effective for protecting local-scale connectivity within the reef lagoon. However, larger scale connectivity between atolls (10s km) created by shark movements remains unprotected. Additionally, cross-taxa movement corridors, or common pathways, are of particular interest considering the metapopulation and management implications of their influence in the connectivity within an ecosystem. Our study further supports the need for larger protected areas to effectively encompass mobile reef predators and the ecological connectivity they maintain [24, 67, 74]. It is crucial that we optimise existing management and conservation strategies to ensure resilience of highly threatened habitats such as coral reefs and marine predator populations which are in decline globally.

## Supplementary Information

Supplementary Material 1

Supplementary Material 2

## Data Availability

Acoustic tracking data can be downloaded from the Australian Animal Acoustic Telemetry Database of the Integrated Marine Observing System (IMOS) housed in the AODN portal (https://portal.aodn.org.au/).
