# Supplementary material for: Marine predator movements create seascape connectivity in remote coral reef ecosystems
Source: Mov Ecol. 2025 Oct 10;13:72. doi: 10.1186/s40462-025-00598-7 (PMC12512746; doi:10.1186/s40462-025-00598-7)
Supplement: Supplementary file 1 — Supplementary Material 1 [file 40462_2025_598_MOESM1_ESM.docx]

## Supplementary Figures


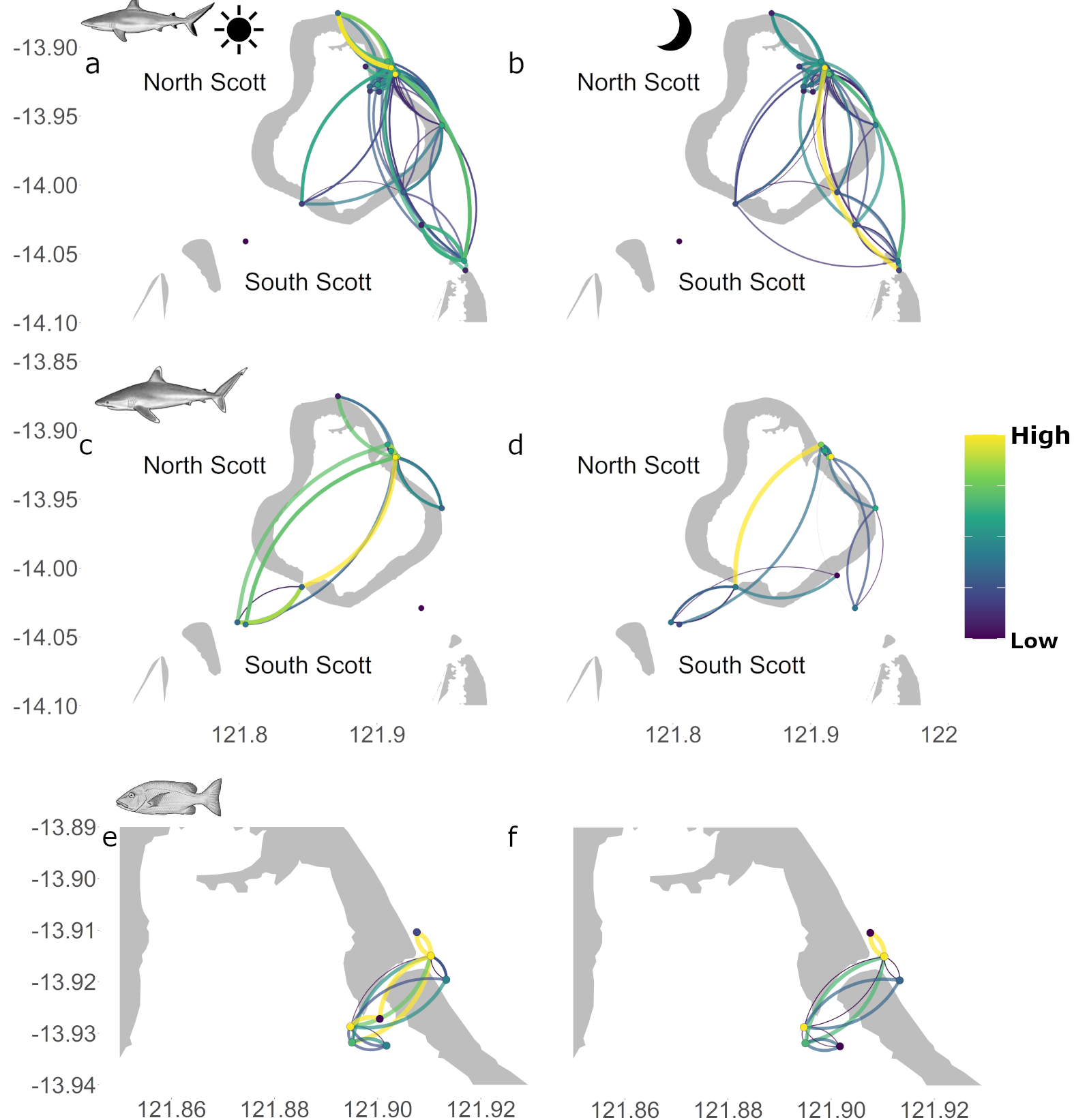


Figure S1 – General networks for grey reef sharks (a-b), silvertip sharks (c-d) and red bass (e-f) at Scott Reef, during the day (a,c,e) and at night (b,d,f). The colour scale indicates values of degree for each node and betweenness for each edge, however they were scaled as high-low within each network to aid visualisation, directionality of edges is represented in a clockwise movement.


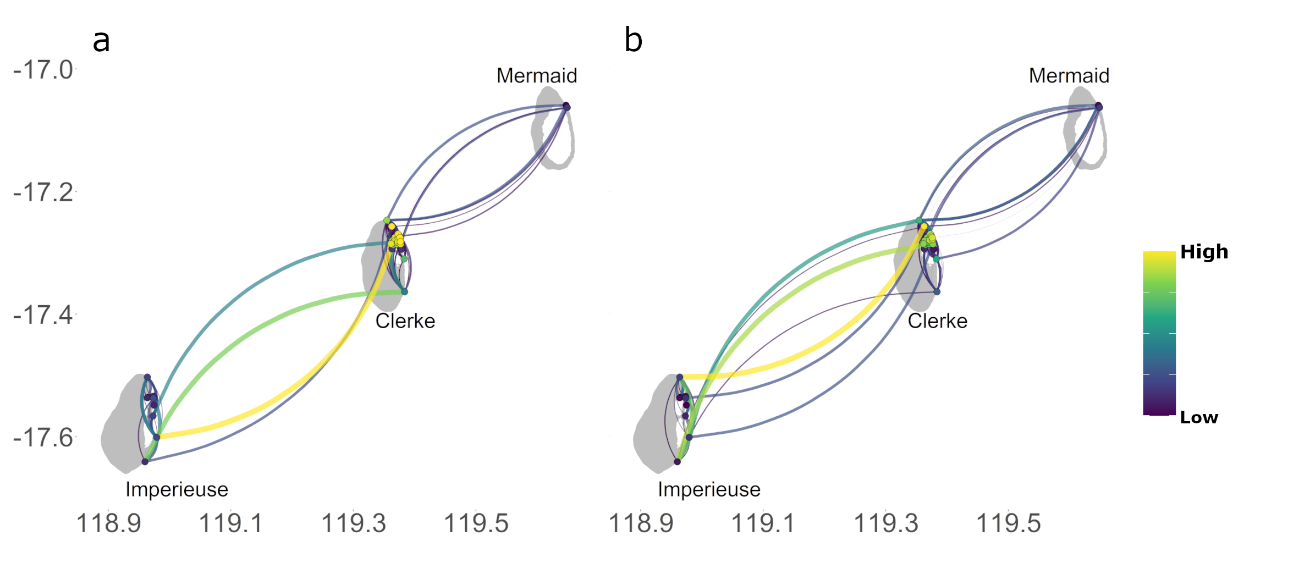


Figure S2. – General networks for grey reef sharks at Rowley Shoals, during the day (a) and at night (b). The colour scale indicates values of degree for each node and betweenness for each edge, however they were scaled as high-low within each network to aid visualisation, directionality of edges is represented in a clockwise movement.


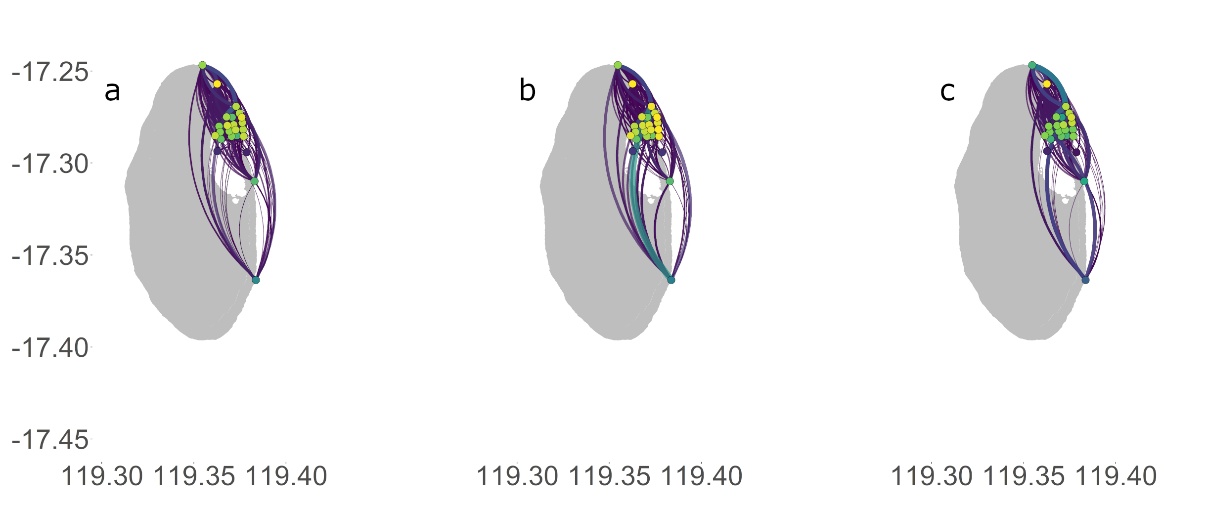

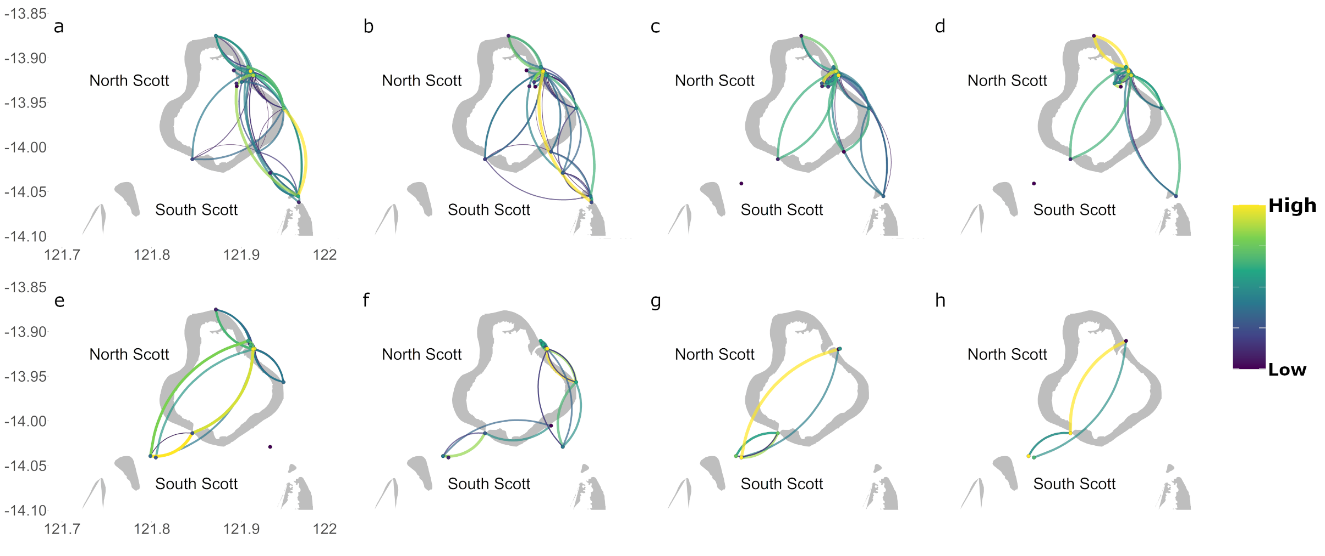


Figure S3. Networks for grey reef sharks at Clerke Reef, Rowley Shoals. Panels indicate full networks (a), day network (b) and night network (c). The colour scale indicates values of degree for each node and betweenness for each edge, however they were scaled as high-low within each network to aid visualisation.


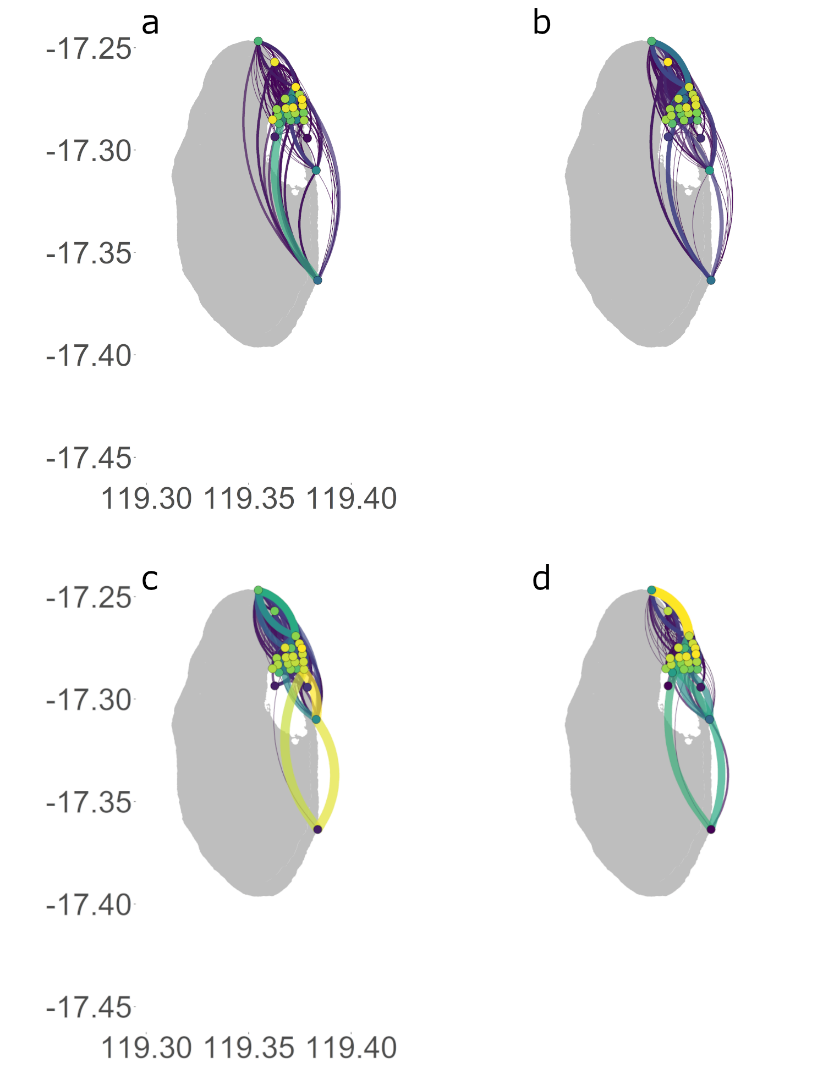

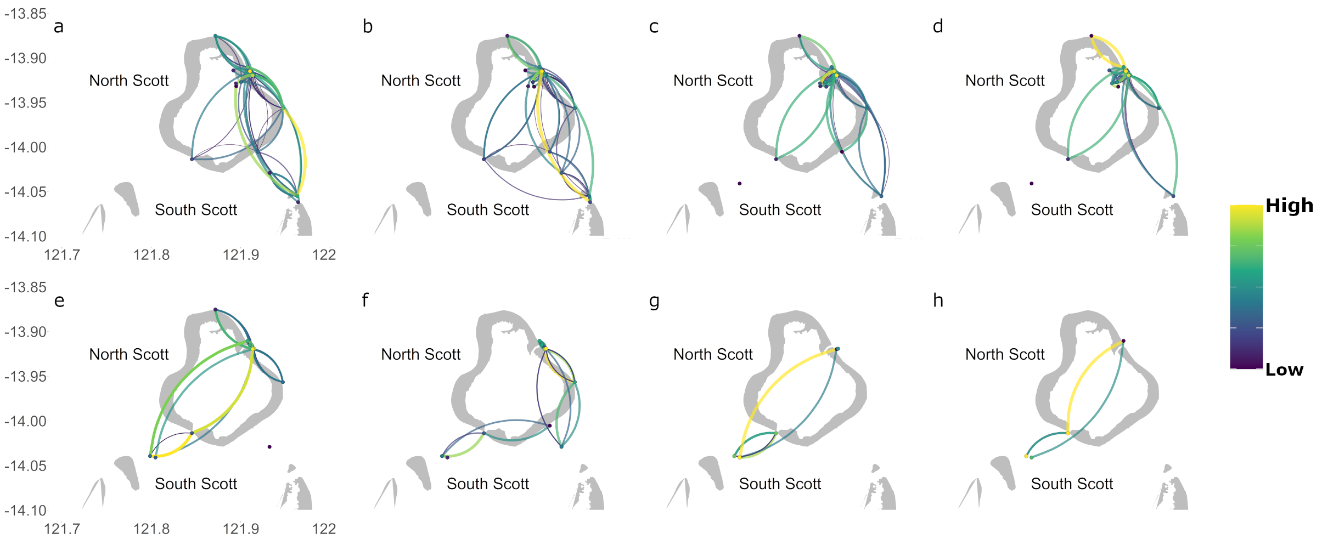


Figure S4. Networks for grey reef sharks at Clerke Reef, Rowley Shoals. Panels indicate female networks (a-b) during the day (a) and at night (b) and male networks (c-d) during the day (c) and at night (d). The colour scale indicates values of degree for each node and betweenness for each edge, however they were scaled as high-low within each network to aid visualisation.


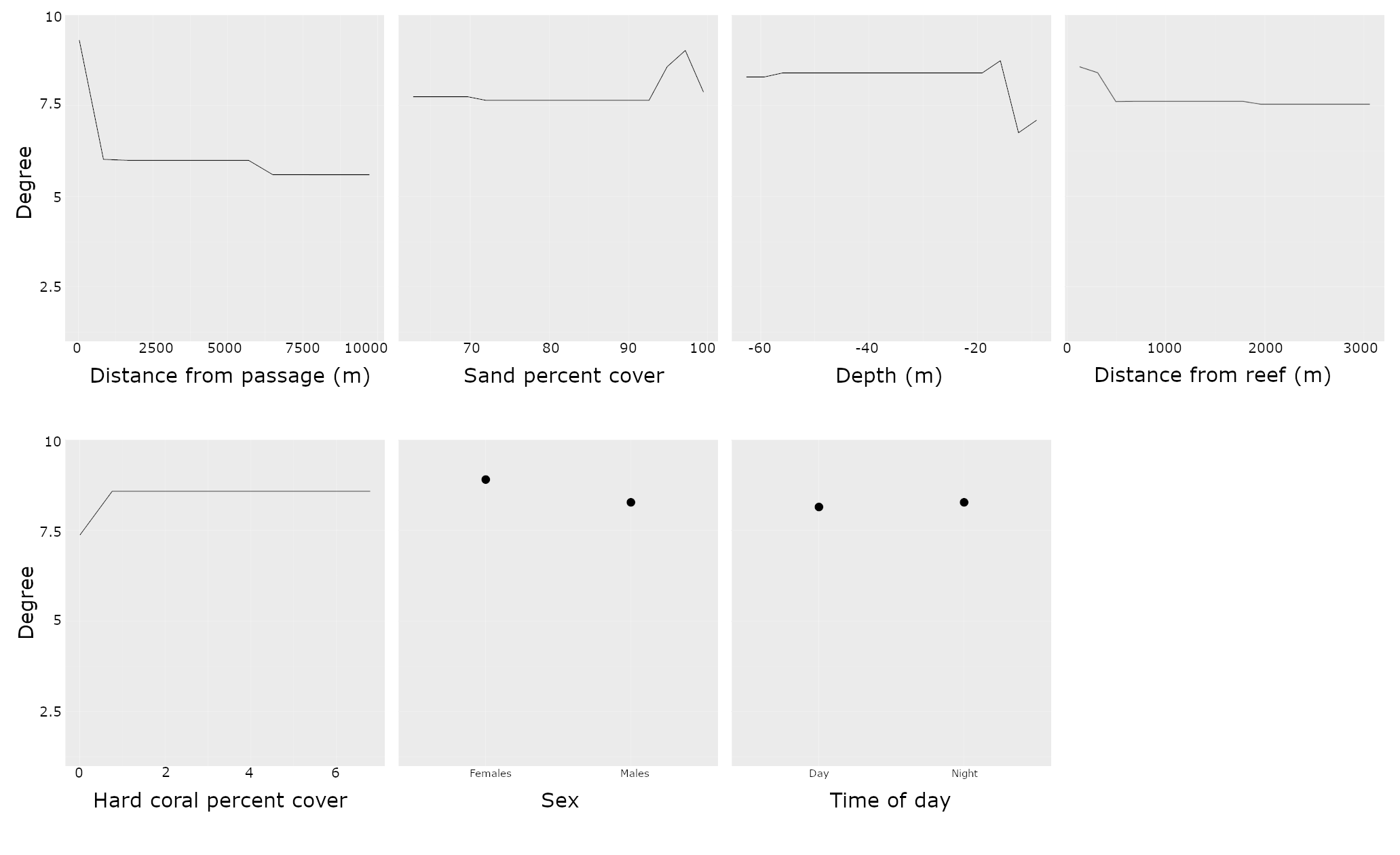


Figure S5. Partial plots from gradient boosted models for covariates explaining centrality measures (degree) of all individual networks of grey reef sharks at Scott Reefs. Details of each explanatory variables are summarised in Table S1 and S2.


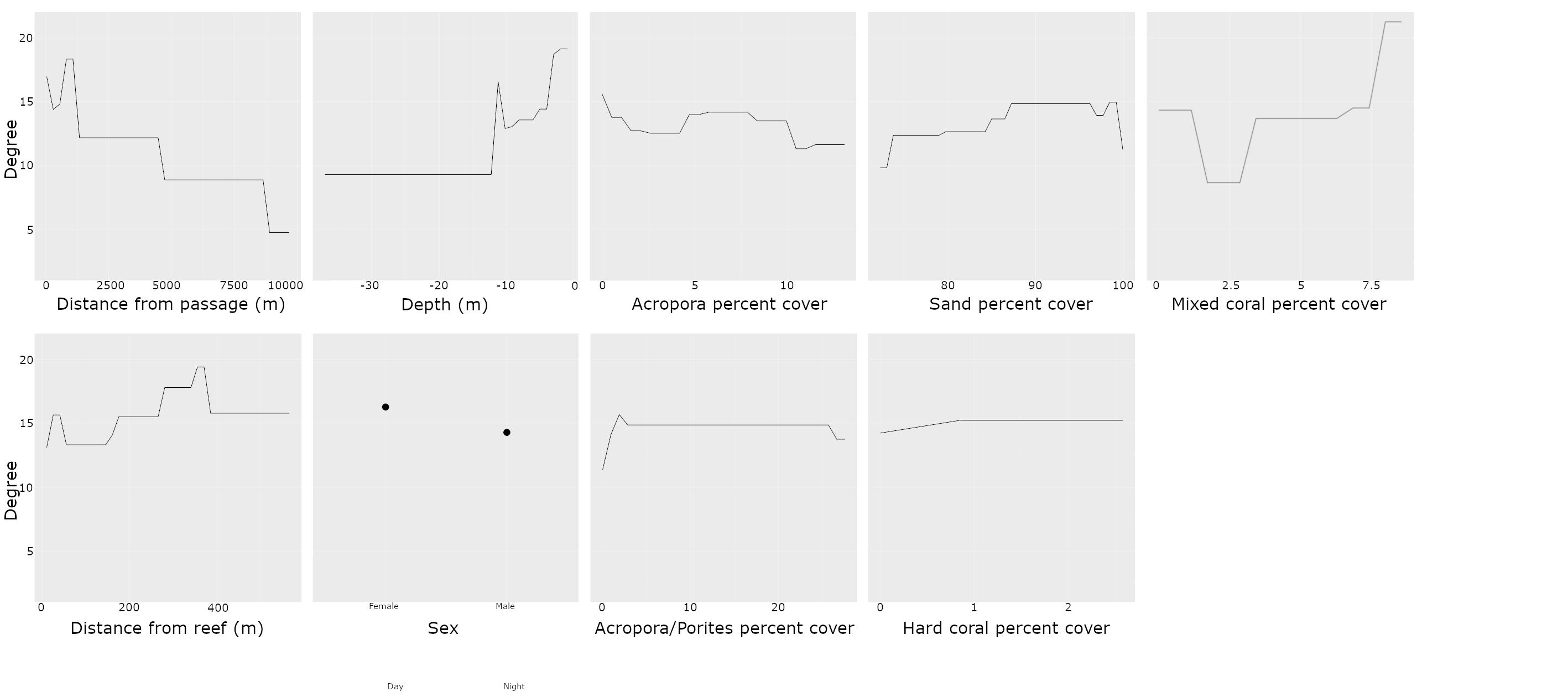


Figure S6. Partial plots from gradient boosted models for covariates explaining centrality measures (degree) of all individual networks of grey reef sharks at Rowley Shoals combined. Details of each explanatory variables are summarised in Table S1 and S2.


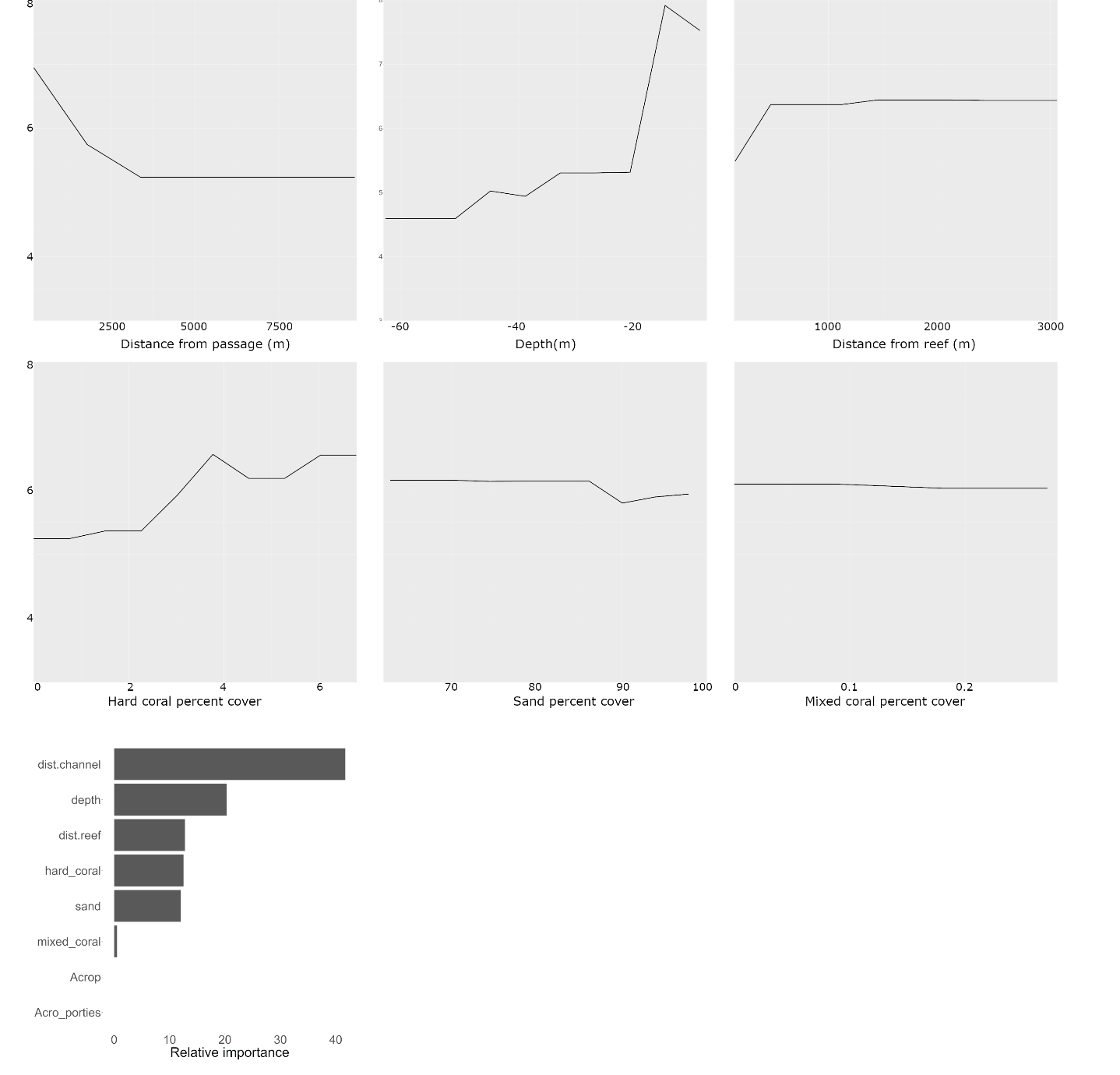


Figure S7. Partial plots and relative importance (bottom) from gradient boosted models for covariates explaining centrality measures (degree) of all individual networks of silvertip sharks at Scott Reef combined. Details of each explanatory variables are summarised in Table S1 and S2.


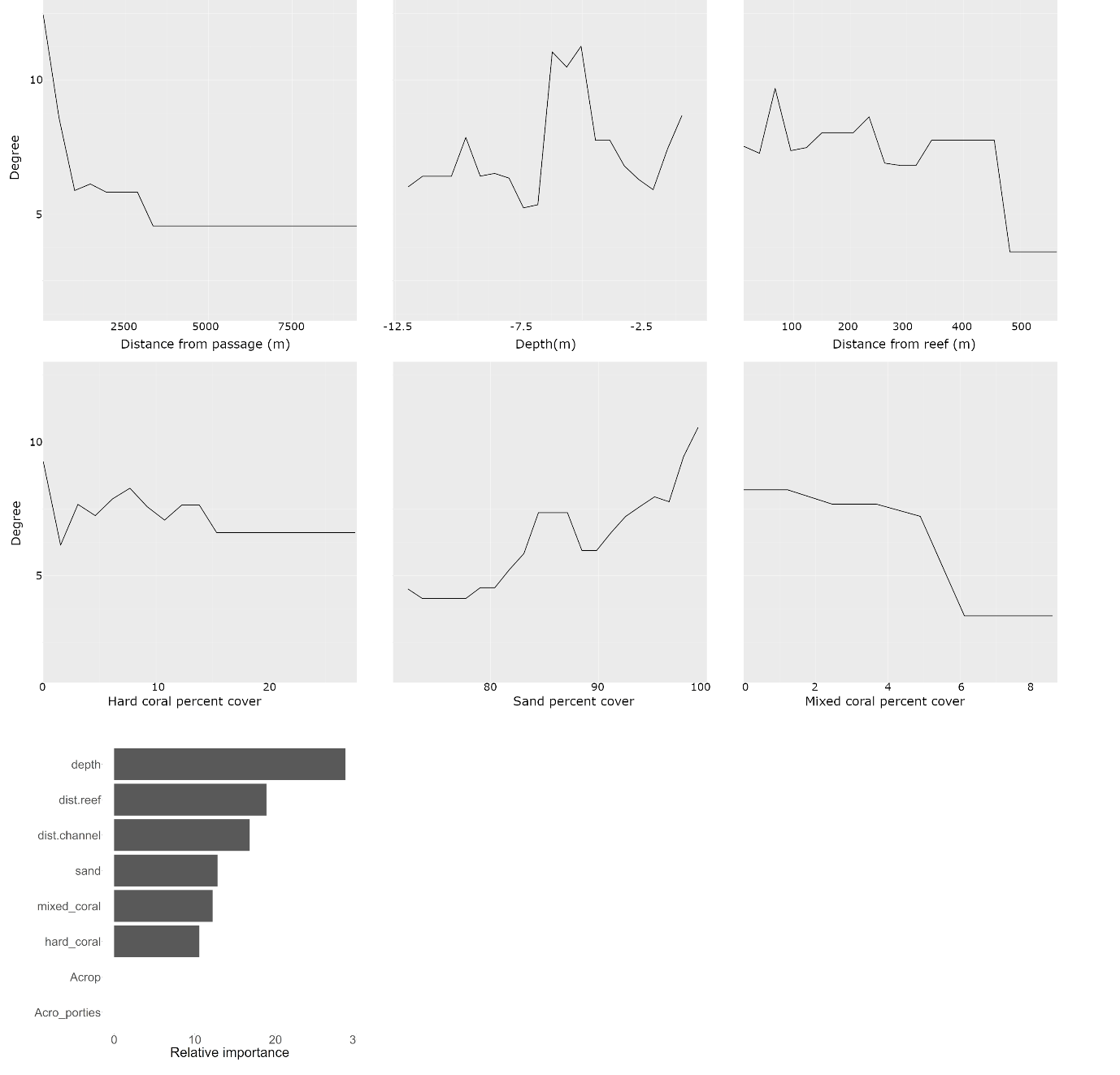


Figure S8 Partial plots and relative importance (bottom) from gradient boosted models for covariates explaining centrality measures (degree) of all individual networks of red bass at Rowley Shoals combined. Details of each explanatory variables are summarised in Table S1 and S2.
