# Supplementary material for: Marine predator movements create seascape connectivity in remote coral reef ecosystems
Source: Mov Ecol. 2025 Oct 10;13:72. doi: 10.1186/s40462-025-00598-7 (PMC12512746; doi:10.1186/s40462-025-00598-7)
Supplement: Supplementary file 2 — Supplementary Material 2 [file 40462_2025_598_MOESM2_ESM.docx]

Table S1. A description of habitats classifications and abbreviations provided by the habitat model.

| **Abbreviation** | **Benthic group classification** |
| --- | --- |
| Acropora | Acropora coral cover |
| Acro_porites | Acropora and Porites cover |
| hard_coral | Foliose hard coral |
| hand | Sand pavement with sparse coral |
| Mixed_coral | Mixed hard and soft coral |

**Table S2.** List of predictors used in gradient boosted models.

| **Predictor** | **Name** | **Description** |
| --- | --- | --- |
| dist.reef | Distance from reef | Distance in metres calculated from the geographic location of each receiver station to nearest reef |
| dist.channel | Distance from channel | Distance in metres calculated from the geographic location of each receiver station to the largest passage in the reef crest |
| Depth | Depth | Water column depth at each receiver station was determined using three complementary data sources; Satellite Derived Bathymetry (Radford et al., 2024), LIDAR, and multibeam data. The LIDAR data was provided by the Royal Australian Navy for Rowley Shoals and by Woodside Energy Limited for North and South Scott Reef. Multibeam data for the South Scott Reef Lagoon came from the Australian Institute of Marine Science and Geoscience Australia. |
| Habitat classification (described on Table S2) | Benthic group classification | Proportion of coverage of the area around each receiver station (300 m radius) |
